# Applying OHSA to Detect Road Accident Blackspots

**DOI:** 10.3390/ijerph192416970

**Published:** 2022-12-17

**Authors:** Zhuang-Zhuang Wang, Yi-Ning Lu, Zi-Hao Zou, Yu-Han Ma, Tao Wang

**Affiliations:** School of Naval Architecture Ocean and Civil Engineering, Shanghai Jiao Tong University, Shanghai 200240, China

**Keywords:** crashes, blackspot identification, optimized hot spot analysis, road traffic safety

## Abstract

With increasing numbers of crashes and injuries, understanding traffic accident spatial patterns and identifying blackspots is critical to improve overall road safety. This study aims at detecting blackspots using optimized hot spot analysis (OHSA). Traffic accidents were classified by their participants and severity to explore the relationship between blackspots and different types of accidents. Based on the outputs of incremental spatial autocorrelation, OHSA was then implemented on different types of accidents. Finally, the performance of OHSA in evaluating the road safety level of the proposed RBT index are examined using a binary correlation analysis (i.e., R^2^ = 0.89). The results show that: (1) The optimal scale distance varies from 0.6 km to 2.8 km and is influenced by the distance of the travel mode. (2) Central cities, with 54.6% of the total accidents, experiences more rigorous challenges regarding traffic safety than satellite cities. (3) There are many types of black spots in vulnerable communities, but in some specific areas, there are only black spots of non-motor vehicle accidents. Considering the practical significance of the above results, policy makers and traffic engineers are expected to give higher attention to central cities and vulnerable communities or prioritize the implementation of relevant optimization measures.

## 1. Introduction

As a common kind of road hazard, traffic accidents can interfere with the long-term operational stability of urban road networks. According to the statistics of the National Bureau of Statistics of China [1], the total number of crashes, property damage and the number of fatalities in China have all increased significantly. The number of crashes in 2020 was 1.3 times than that of 2015. Some developed countries also face the same challenges. In the United States, the number of fatalities caused by traffic accidents in 2010 was 32,999, while the number of fatalities caused by traffic accidents in 2020 was 38,824 [2]. In the past decade, the number of fatalities has increased by 17.7 percent. Traffic accidents not only endanger people’s lives, but also lead to severe traffic congestion and increased pollutant emissions. Therefore, reducing the number of traffic accidents and improving traffic safety play an important role in the urban economy, inhabitant trips and social stability [3]. Most scholars have employed advanced statistical models and cutting-edge machine learning methods to predict the number of crashes and analyze accident severity [4,5,6]. Policy makers still need to take a detailed look at the spatial clustering patterns of crashes to support the implementation of relevant optimization measures.

Regional economic disparity leads to the uneven development of traffic infrastructure, road design and traffic management in different regions [7,8]. Under this circumstance, traffic accidents also tend to happen in certain road segments of one city. Blackspots (i.e., accident-prone road sections) are defined as the road segments or areas where traffic accidents are highly concentrated [9]. Previous studies have shown that the increase in traffic accident fatalities is not only related to the increase in vehicle ownership, but also to inefficient and inaccurate traffic safety improvement measures [10,11]. Identifying blackspots can tell traffic managers how accidents are spatially distributed, whether different types of accident blackspots overlap, and where the city’s vulnerable communities are. Moreover, the optimization measures implemented for the whole road network rather than the key areas can easily exceed the cost limit. However, cost savings can be achieved by implementing optimization measures only for blackspot areas [12]. The money saved can be invested in infrastructure construction, education and poverty alleviation to narrow the gap between rich and poor and improve urban equity. Therefore, it is of great significance to identify accident blackspots for improving urban traffic safety [13,14,15]. 

Based on black spot identification results, optimization measures can be accurately implemented by traffic engineers. The traditional method is to calculate the number or rate of traffic accidents on a road segment [16]. If the statistic value on a road segment is greater than the pre-defined threshold, it is considered to be an accident black spot [14]. Therefore, unreasonable threshold setting could lead to inaccurate black spot identification. To solve this problem, a wide variety of statistical methods has been proposed to analyze historical traffic accident data [4,17]. Typical statistical methods include logit models [18], lognormal models [19], the Poisson model [14], Fault Tree [13] and the Bayesian model [20]. Although these methods have a clear functional form, they require that historical traffic accident data obeys strict predefined distribution [21]. If the assumptions do not hold, the model estimation results can be biased and erroneous [21]. Moreover, traditional statistical methods cannot capture the spatial autocorrelation of traffic accident features [22]. To further improve the accuracy of blackspot identification, an increasing number of scholars focus on spatial data mining technology.

With the development of geographic spatial data processing technology and digital maps, spatial data mining has become an active technology in traffic safety research, which is trying to answer the questions of “why” and “where” crashes happen. In this field, hotspot mapping techniques are the most common and effective method. Existing hotspot mapping techniques such as point mapping, kernel density estimation (KDE), K-means clustering and thematic mapping focus only on target feature density [23]. Standard deviational ellipses (SDE) were used earlier to make effective descriptions of areal point data [24]. Using a standard deviation ellipse to identify blackspots requires sufficient evidence that crash events comply with the elliptical distribution. Most research has relied on the KDE technique and K-means clustering. For example, Anderson [25] proposed a methodology using Geographic Information Systems (GIS) and KDE to investigate the spatial clustering of road accidents. In his study, K-means clustering is applied to find the most similar pair of clusters. Similarly, KDE and K-means clustering are also used by Ryder et al. to profile road accident hotspots in vehicle decision-support systems [26]. However, when the data does not exist in a uniform planar space, the KDE model is biased because of possible asymmetry, curvature, or boundary effects in the data [27]. In other words, the conventional KDE model is not so applicable in a linear space, such as a road network [28]. To overcome these limitations, some researchers proposed various KDE extension modes, including network-based KDE [29] and spatio-temporal KDE [30]. Although KDE extension modes overcome the limitation of planar space, they still cannot provide robustness that can be measured by a statistical index. In fact, the probability of the occurrence of different types of accidents is not determined. The ideal model results need to provide the confidence level at which each spatial unit is identified as a hotspot in the produced map.

Optimized Hot Spot Analysis (OHSA) is essentially a clustering method that can identify statistically significant clusters of target features [31]. To reflect the observed spatial clustering of high/low values, OHSA is able to output z-score, *p*-value, and confidence interval fields by using the Getis-Ord Gi* statistic [32]. To be a hot/cold spot with statistical significance, the spatial unit not only needs to have a high/low number of traffic accidents but also needs to be surrounded by other units with high/low values as well [10]. The local sum of the number of traffic accidents in a spatial unit and its neighbors is compared with the sum of the number of traffic accidents in all spatial units on a proportional basis [33]. Wang et al. [23] proved that it is powerful in identifying the linkage between target crime and related factor after the implementation of the crime hotspot analysis. Lu et al. [34] first applied OHSA to landslide hotspot detection. So far, OHSA has been used in a variety of fields, including disaster detection [35], crime hotspot identification [23], demography [36], epidemiology [37] and biology [38]. However, relatively few studies applied OHSA to blackspot identification.

The new method used in this study can capture the spatial clustering patterns and provide statistical significance for every space unit. This article uses the crash dataset from the Liangshan Yi Autonomous Prefecture in Sichuan Province, China, and employs the OHSA method to explore blackspots for different types of accidents. The contributions of our study are as follows: First, our method demonstrates the viability of OHSA to explore the space distribution of blackspots. This is an effective approach that can help traffic managers and police officers understand the spatial clustering patterns of traffic accidents. Second, compared to regression models, our model can help urban planners to identify vulnerable urban communities with overlapping blackspots of different types of accidents. Third and finally, the proposed RBT index (i.e., the ratio of the number of spatial units occupied by blackspots to the total number of spatial units) is an alternative measurement from existing indicators of urban traffic safety level.

## 2. Methodology

**Figure 1:**
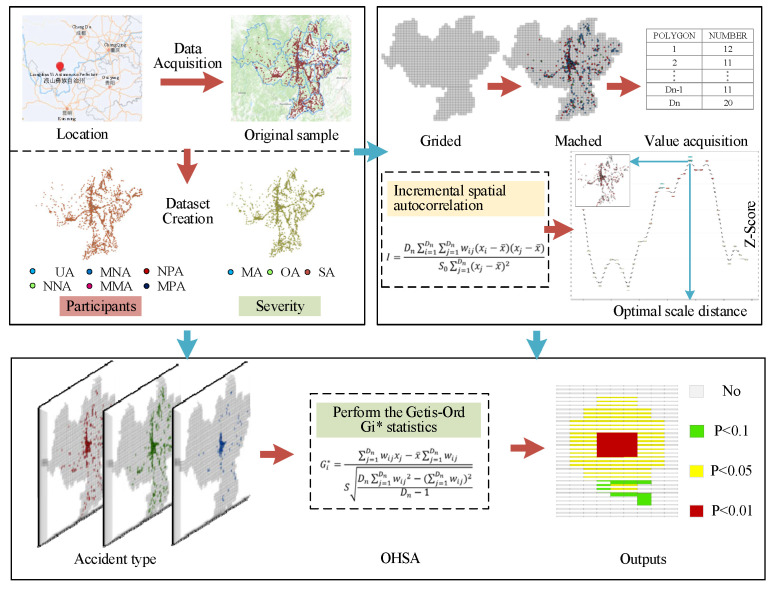
The flowchart of the proposed method.

To explore the relationships between the spatial distribution of blackspots and crash types, it is necessary to classify traffic accidents into different types. The classification of road accident hotspots is generally based on available data relating to the accident itself [25]. According to accident participants, traffic accidents can be classified into six main categories, namely unilateral accidents (UA), motor vehicle accidents (MMA), motor vehicle and non-motor vehicle accidents (MNA), motor vehicle and pedestrian accidents (MPA), non-motor vehicle accidents (NNA), and non-motor vehicle and pedestrian accidents (NPA). In addition, traffic accidents can be classified into three main categories according to the accident severity, namely minor accidents (MA), ordinary accidents (OA) and serious accidents (SA). It is worth noting that the total number of crashes under different classification methods remains equal to the original sample size. OHSA was then carried out on all the above accident types to identify their blackspots. The approach was implemented in Environmental Systems Research Institute ArcGIS Pro [33]. The flowchart of OHSA is illustrated in Figure 1.

Before applying OHSA to identify blackspots, it is necessary to define a suitable analysis distance as the input of scale distance for the Getis-Ord Gi* statistics. The ideal analysis range should appropriately reflect the inherent characteristics of spatial units to be analyzed. The more realistic the model of how features interact with each other in space, the more accurate the results [33]. When this parameter cannot be measured effectively, a valid approach is to consider the spatial relationships between the analysis distance and clustering patterns of features. The incremental spatial autocorrelation tool measures the spatial autocorrelation of a series of distance increments and optionally creates a line chart of those distances and their corresponding z-scores that reflects the degree of spatial clustering [39]. The optimal scale distance corresponds to the statistically significant peak z-scores. Taking the optimal scale distance (i.e., peak distances) as the input of OHSA can make the results more accurate. Generally, the Global Moran’s I statistic is used to measure the degree of spatial autocorrelation:

(1) $$I=\frac{D_{n}\sum_{i=1}^{D_{n}}\sum_{j=1}^{D_{n}}w_{ij}\left(x_{i}-\bar{x}\right)\left(x_{j}-\bar{x}\right)}{S_{0}\sum_{j=1}^{D_{n}}{\left(x_{j}-\bar{x}\right)}^{2}}$$

(2) $$S_{0}=\overset{D_{n}}{\underset{i=1}{\sum }}\overset{D_{n}}{\underset{j=1}{\sum }}w_{ij}$$

where, $D_{n}\;$ represents the total number of spatial units; $x_{i}$ and $x_{j}$ represent the number of a certain type of traffic accidents in spatial units i and; $\bar{x}$ is the average number of traffic accidents in all spatial units; $w_{ij}$ is the spatial weight between unit i and unit j. The spatial weight matrix can be measured according to the adjacency or distance criterion; $S_{0}$ is the sum of spatial weights, which can be calculated by Equation (2). Based on the results of the Global Moran’s I, the z-score that determines the significance level of spatial autocorrelation can be calculated according to the following equation:

(3) $$Z=\frac{I-E\left(I\right)}{\sqrt{Var\left[I\right]}}$$

(4) $$E\left(I\right)=\frac{-1}{n-1}$$

(5) $$Var\left(I\right)=-E\left[I^{2}\right]-E{\left[I\right]}^{2}$$

where, $E\left(I\right)$ and $Var\left[I\right],\;\mathrm{respectively}$, represent the expected value and the variance of the Global Moran’s I. Based on the value of z-score, it is possible to estimate the degree of spatial autocorrelation. In this study, the scale distance with the highest z-score is selected as the appropriate value of the distance band for OHSA.

OHSA calculates the Getis-Ord Gi* statistics for the crash density inside a spatial unit (polygon) and determines their statistical significance using the z-score and *p*-value. Both of them are measures of statistical significance and are used to determine whether the null hypothesis is rejected. The combination of the z-score and *p*-value can effectively capture the difference results caused by the random distribution, because the high and low value spatial clustering mapped by the them is more pronounced than expected in the random distribution with the same value. In addition, the multiple tests and spatial dependencies of the Gi* statistical results can be automatically corrected using the false discovery rate correction method. For each single spatial unit i, the Gi* index within a scale distance d can be estimated as:

(6) $$G_{i}^{*}\left(d\right)=\frac{\sum_{j=1}^{D_{n}}w_{ij}x_{j}-\bar{x}\sum_{j=1}^{D_{n}}w_{ij}}{S\sqrt{\frac{D_{n}\sum_{j=1}^{D_{n}}w_{ij}{}^{2}-{\left(\sum_{j=1}^{D_{n}}w_{ij}\right)}^{2}}{D_{n}-1}}}$$

(7) $$S=\sqrt{\frac{1}{D_{n}}\overset{D_{n}}{\underset{j=1}{\sum }}x_{j}{}^{2}-{\bar{x}}^{2}}$$

where, the value of $G_{i}^{*}$ index is the z-score. The statistical significance of the z-score is the multiple of the standard deviation. Therefore, the conditions under which a spatial unit is identified as a hotspot at 99%, 95% and 90% confidence levels are z-score > 2.58, 1.96 < z-score < 2.58, and 1.65 < z-score < 1.96, respectively. Similarly, the conditions under which a spatial unit is identified as a coldspot at 99%, 95% and 90% confidence levels are z-score < −2.58, −2.58 < z-score < −1.96, and −1.96 < z-score < −1.65, respectively. In other cases, the spatial unit is not significant. If a feature has a high z-score and a small *p*-value, it indicates a spatial cluster of high values [33]. If the z-score is low and negative and the *p*-value is small, there is a spatial cluster of low values [33].

The final step is to visualize the z-score of each spatial unit corresponding to a particular type of traffic accident on the map. In this study, we define the blackspot as a hotspot at a 99% confidence level (i.e., *p* < 0.01, z-score > 2.58). 

## 3. Study Area and Dataset

The study area is Liangshan Yi Autonomous Prefecture located in the southwest of Sichuan province, China, covering a total area of 60,400 square kilometers [40]. The reasons for selecting the study area are listed as follows: (1) Sichuan is a typical inland province that could represent the development level of Southwest China. Liangshan Yi Autonomous Prefecture, governing two cities and 15 counties, is the largest autonomous prefecture in Sichuan province. By the end of 2021, the total resident population of the prefecture was 4.874 million, the urbanization rate was 38.66%, and the road mileage of the prefecture reached 27,600 km [40]. The selection of Liangshan Yi Autonomous Prefecture as the study city has huge practical significance. (2) The prefecture has a perfect traffic law enforcement system, especially traffic safety management. In order to achieve better road safety management, the local traffic bureau has a professional technical team responsible for handling unexpected traffic accidents in real time. Technical team members are required to make a detailed record for each traffic accident, including time of day, accident location, accident description, accident participants and accident severity.

**Figure 2:**
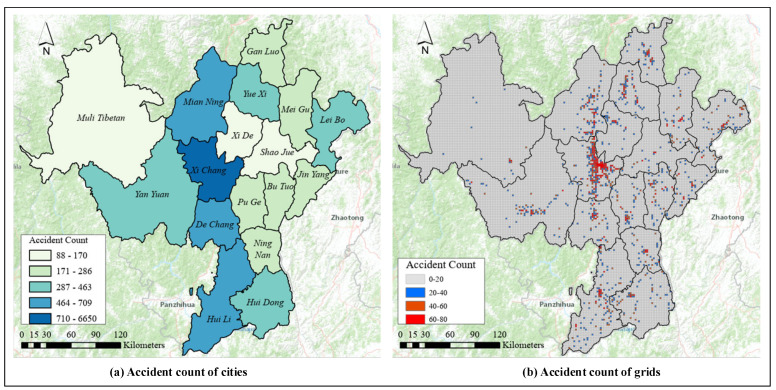
Distribution of traffic accidents: (**a**) accident count of cities, (**b**) accident count of grids (200 m × 200 m).

As a result, we obtained the local traffic accident records from 1 January to 31 December 2019. To control the data quality, the 3-sigma principle was used to eliminate outliers from the original data set. After the data cleaning, a total of 12,187 valid samples were obtained. Figure 2a shows the number of traffic accidents in each area. In terms of the spatial distribution density of points, the central city (i.e., Xichang) has the highest number of accidents, with 54.6% of the total accidents. In contrast, Muli Tibetan Autonomous County has the largest area but the least traffic accidents. To investigate the spatial differences of traffic accidents, this study used the rasterization method to split each area. The latitude and longitude information of the study area were extracted using ArcGIS software to split polygons into grids with equal spatial intervals (i.e., 200 m × 200 m). Correspondingly, the traffic accident concentration in the study areas could be normatively counted and displayed in the grids. Figure 2b shows the gridded study area. The number of traffic accidents in the red grid is the largest. Taking Xichang as an example, the accident features were matched to the corresponding grids. Subsequently, the number of traffic accidents of each grid was integrated into one new data set for feeding into OHSA to identify blackspots. It can be seen that the shape of the blackspot area is based on the grid shape and size.

Table 1 shows a summary of the crash dataset. According to the classification of accident participants, the number of motor vehicle accidents (MMA) is the largest, with a total of 8012, accounting for 65.7% of the annual total; the number of non-motor vehicle and pedestrian accidents (NPA) is the lowest, with 215 accidents, accounting for 1.8% of the annual total. When categorized by accident severity, the number of minor accidents (MA) is the largest, with a count of 11,456, accounting for 94% of the year’s total; and the number of serious accidents (SA) is the lowest, with a count of 38, accounting for 0.31% of the year’s total. 

## 4. Results and Discussion

**Figure 3:**
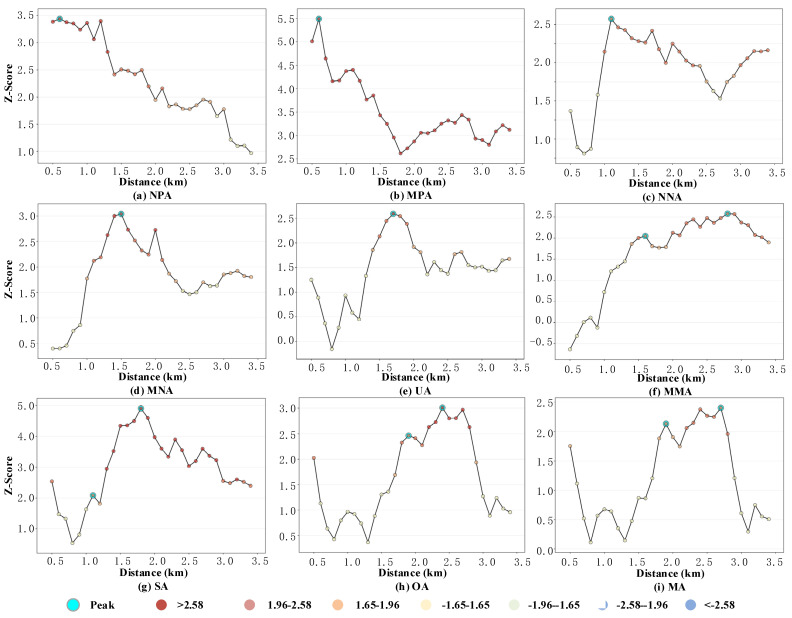
The results of incremental spatial autocorrelation for different types of accidents.

Esri ArcGIS is the most widely used GIS with many novel and practical geographic data mining tools, including the incremental spatial autocorrelation and OHSA [10,33,39]. Based on the classification of traffic accidents, the Global Moran’s I index is calculated using incremental spatial autocorrelation to find the scale distance with the highest z-score (i.e., the optimal scale distance). Then, the optimal scale distance and the accident spatial point of the target type are used as the input of the OHSA model to implement the Getis-Ord Gi* statistics, as seen in Figure 3. To find the optimal scale distance for each type of accident, the analysis distance increases uniformly from 500 m to 3.5 km with a step of 100 m. 

Figure 3 shows the relationships of scale distances and z-scores for each type of accident. The evidence that at least one peak point exists on the curve for each type of accident means that it is reasonable to fix the analysis range between 0.5 km and 3.5 km. When classifying accidents by participants, as seen in Figure 3a–f, the optimal scale distance varies from 0.6 km to 2.8 km, with a mean value of 1.38 km. When classifying accidents by severity, as seen in Figure 3g–i, the optimal scale distance varies from 1.8 km to 2.7 km, with a mean value of 2.3 km. These results show that the optimal scale range is different for different types of accidents. Another interesting finding is that the optimal scalar distance is positively correlated with the distance of resident travel modes. For example, for accidents involving pedestrians, that is, NPA and MPA, the distance corresponding to the highest z-score is 0.6 km. This distance is obviously significantly smaller than the mean value of the same category (i.e., 0.6 < 1.38) and is strongly correlated with the travel distance of walking. Another possible reason may be that accidents involving pedestrians are often concentrated at intersections. Similarly, the optimal scalar distance increases significantly with the involvement of motor vehicles, namely 1.1 km (NNA) < 1.5 km (MNA) < 2.8 km (MMA), due to the fact that the distance travelled by a car is much longer than by walking or cycling. This means that if a suitable distance parameter is to be chosen for traffic accident analysis, the accident type should be fully considered. The ideal scalar distance varies with the type of accident and is influenced by the distance of the travel mode. Simply taking the entire accident dataset as the model input without considering the classification may result in unreasonable distance bands, which further leads to insignificant feature identification or the loss of data continuity.

**Figure 4:**
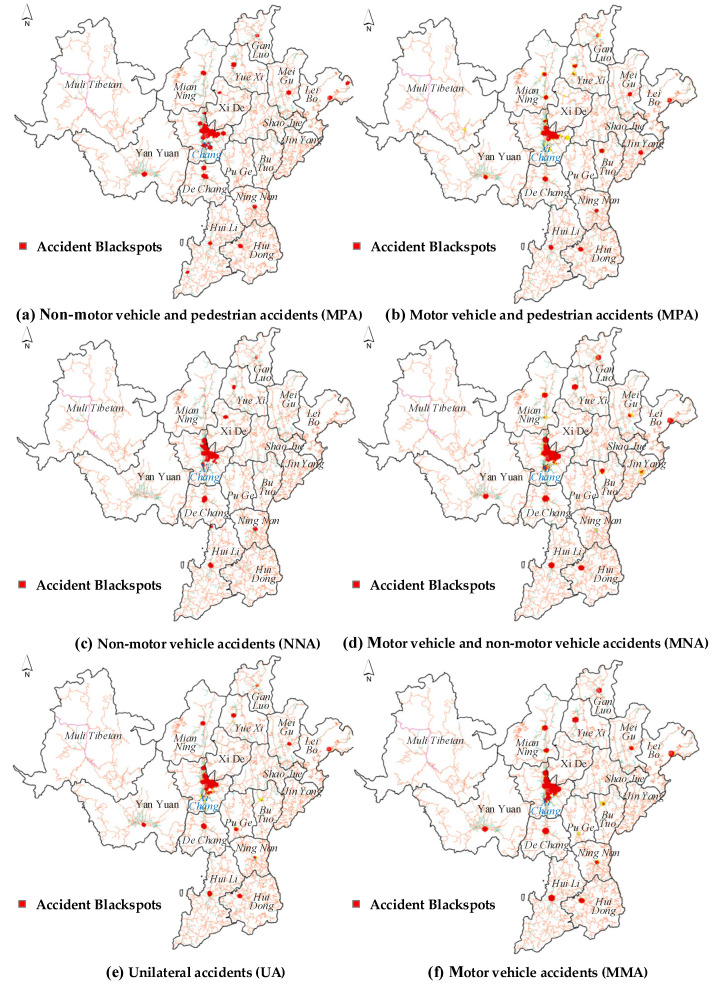
OHSA identification results for classifications by accident participants.

After performing OHSA, all spatial units can be classified into seven patterns, namely spatial hotspots at 99% confidence level (*p* < 0.01), spatial hotspots at 95% confidence level (*p* < 0.05), spatial hotspots at 90% confidence level (*p* < 0.1), insignificant spatial units, spatial coldspots at 99% confidence level (*p* < 0.01), spatial coldspots at 95% confidence level (*p* < 0.05), and spatial coldspots at 90% confidence level (*p* < 0.1). Among these patterns, we focus on spatial hotspots at 99% confidence level (i.e., blackspots), as mentioned earlier. However, compared to other patterns, the number of spatial hotspots at 99% confidence level is not so large, which may cause the visualization results to be less easily understood. In order to make these features more clearly mapped on the map, the insignificant spatial units are transparently shown, and the hotspots at three confidence levels are represented by a red, yellow and green polygon, respectively, as seen in Figure 4. The distribution of red grids can be clearly observed, while the yellow and green grids are not so easily found. This indicates that the spatial hotspots at 99% confidence level (*p* < 0.01) occupy a large portion of the spatial units with statistical significance.

Such promising conclusions can be attributed to three factors. First, OHSA has a clear advantage in identifying blackspots. Spatial clustering is able to describe the excess of crashes in geographic spaces with higher or lower spatial densities, and OHSA is such a method that uses Getis Ord Gi* statistics to capture the clustering nature of crash points [34,41]. Compared with traditional regression methods, OHSA does not require strict prior distribution [42]. Second, OHSA has a powerful mechanism for handling location outliers in data sets. Location outliers can have a large impact on spatial statistics [10]. To mitigate the impact of outliers, OHSA calculates the average distance from each feature to its nearest neighbor and examines the distribution of all these distances [39]. If a feature is more than three standard deviations away from its nearest non-coincident neighbor, it is considered a location outlier [33]. Third, in the case of traffic accidents, blackspots can only be captured by OHSA if they also have a potential tendency to cluster spatially. Traffic accidents appear to occur at or near specific road segments where they may have occurred in the past and can therefore be spatially discontinuous and mapped in clusters. Compared with the traditional clustering method [28,29], OHSA can give a certain confidence interval for the clustering pattern of each spatial unit. Therefore, OHSA can clearly indicate the existence of blackspots. In other words, OHSA can identify statistically significant spatial clusters for the blackspot detection.

Figure 4 shows the OHSA results classified by accident participants. The central city contains the highest number of blackspots (i.e., red polygon) out of the 17 areas. Moreover, blackspots for each accident type could be found in the central city. This means that road traffic safety in the central city faces a greater challenge than that in satellite cities. The findings match those observed in previous studies [43]. A possible explanation for this might be that the road environment in central cities is much more complex, with a large number of people, businesses and economic activities involved [44]. The density of population, employment and land use play an important role in the geographical distribution of traffic accidents [45]. When these factors interact with one another, their interaction can become complex. Various external factors, such as environmental and social factors, put great pressure on the local road network and raise traffic safety concerns [43]. Compared to the central city, Yuexi, Huili, Dechang, Huidong, and Ganluo do not have the same range of accident blackspots as Xichang, while they still have a wide variety of accident blackspot types. This implies that these cities may only have a few areas where the road environment is as complicated as in the central city. On the contrary, hardly any blackspots can be found in Shaojue, Puge and Muli Tibetan Autonomous County. Regardless of the differences in traffic demand, these cities may have a higher level of road traffic safety. In addition, some cities would only have fixed types of blackspots. For example, Xide only has blackspots where non-motor vehicles are involved (i.e., NPA and NNA). This means that non-motor vehicles are more likely to be responsible for traffic accidents in Xide. Therefore, in order to improve the overall road traffic safety in Xide, policy makers and traffic engineers should enhance the local non-motor vehicle management.

In terms of the location of accidents, the experiment provides an interesting finding: different types of blackspots in a city tend to occur at specific locations or areas. For example, the blackspots of NPA, MPA, MNA, UA, and MMA all appear in the southern part of Yanyuan country. Similarly, the north-central part of the central city is the location where different types of blackspots are found frequently. Our findings are in accord with previous studies indicating that there are potential vulnerable areas in the city which can easily become a place of concentration for various types of traffic accidents [22]. Indeed, the essence of travel is the competition between different types of travel modes for limited urban road resources. Traffic accidents are a dismal result of this competition. For some vulnerable areas in cities, they usually become a concentration of blackspots once they cannot afford the intense traffic competition. Therefore, to enhance road traffic safety, another important conclusion is that policy makers and traffic engineers should pay more attention to these vulnerable communities. The application of OHSA to identify vulnerable communities is very meaningful for comprehensively improving the level of urban traffic safety.

**Figure 5:**
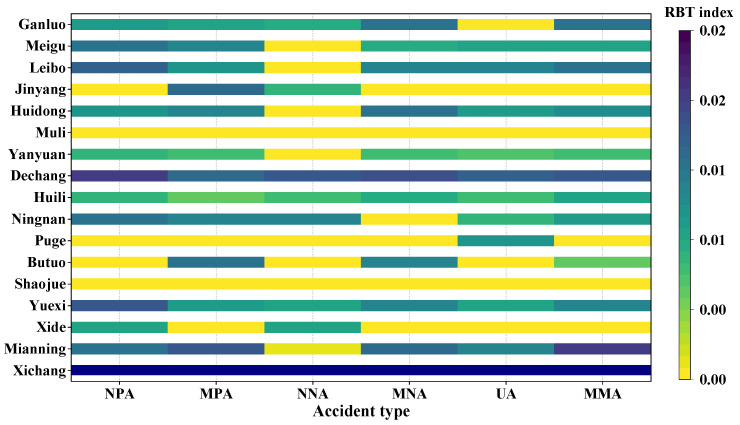
A summary of the RBT index.

Based on the outputs of OHSA, the ratio of the number of spatial units occupied by blackspots to the total number of spatial units (i.e., RBT index) in each city can be calculated as follows:

(8) $$RBT_{c,v}=\frac{B_{n}^{c,v}}{D_{n}^{c,v}}$$

where, $D_{n}^{c,v}$ represents the total number of spatial units under the crash type v in city c; $B_{n}^{c,v}$ is the number of spatial units occupied by blackspots of the crash type v in city c. Therefore, the larger the $RBT_{c,v}$ is, the more widely distributed the blackspots of one certain type of crash is in the city. Accordingly, the level of road traffic safety in the city is worse. Figure 5 is plotted to depict the RBT index of each city under different types of accidents. It can be seen that the central city has the highest RBT value regardless of the types of accidents. This means that the central city (i.e., Xichang) experiences more challenges in terms of road traffic safety than satellite cities. In addition, it is also clear in which cities certain types of accidents are more likely to be clustered according to Figure 5 For example, in Xichang and Yuexi, serious accidents are found in a larger blackspot area than in other areas. This finding indicates that only the implementation of specific measures for certain types of accidents in some cities can effectively reduce the number of traffic accidents.

**Figure 6:**
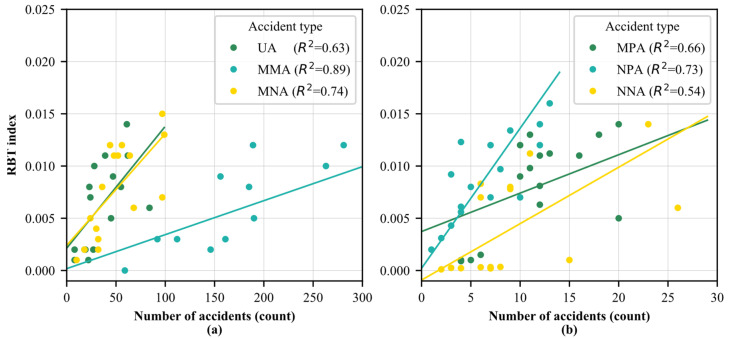
Correlation between the RBT index and the number of accidents: (**a**) correlation analysis of UA, MMA and MNA, (**b**) correlation analysis of MPA, NPA and NNA.

To verify the performance of this new indicator in evaluating regional road safety level, this study performed a binary correlation analysis. As illustrated in Figure 6, the correlation analysis was performed between the RBT index and the number of accidents. The RBT index showed a positive correlation with the number of accidents. Similar results can be obtained from the binary correlation analysis results of six accident types. This finding means that in addition to representing the spatial aggregation degree of a certain type of traffic accident, the accident number can also be reflected by the RBT index to a certain extent. However, there was a weaker positive correlation between the RBT index and the number of accidents (R^2^ = 0.54) in NNA than other accident types. It could be speculated that the black spot distribution of NNA is sparser than that of other accident types. As discussed above, the black spot area of NNA is likely to appear in specific streets, especially in cities where non-motor vehicles are the main mode of travel. Meanwhile, the number of accidents in MMA showed the highest correlation (R^2^ = 0.89) with the RBT index, because the black spots of MMA were widely distributed and the number of MMA was absolutely dominant. Accordingly, the RBT index in one area could provide a reflection on the road safety level.

## 5. Conclusions

With increasing numbers of crashes and injuries, policy makers and traffic engineers over the world are committed to improving road safety. While many studies have targeted this issue by establishing regression models or machine learning models to analyze the factors affecting the number and severity of crashes, policy makers and traffic engineers may still need a detailed identification of blackspots for the implementation of optimization strategy. While there is research showing that drivers become accustomed to the presence of black spots and start to ignore the presence of road signs, the optimization measures implemented in accident black spot areas are not only limited to the installation of road signs, but also include road reconstruction, the deployment of police and installation of automatic law enforcement equipment. Few studies have shown that drivers have abnormal behaviors under the condition of implementing other optimization measures in a black spot area. Therefore, this study focuses on the identification of black spot areas of all types of accidents.

This study proposes a novel method and subsequently evaluates its potential for blackspot detection. First, traffic accident records from 1 January to 31 December 2019 were obtained from the traffic management department of Liangshan Yi Autonomous Prefecture. Second, the original crash dataset was classified by accident participant and accident severity to explore the relationship between the spatial distribution of blackspots and different types of traffic accidents. Thirdly, incremental autocorrelation was implemented to automatically determine the optimal scalar distance for each type of accident. Fourth, the OHSA method was applied to analyze the spatial clustering trends for each type of accident. In OHSA, the z-score (standard deviation) and *p*-value (independent probability) were used as the outputs to map the clustering patterns of spatial units on the map. The OHSA method, based on Getis Ord Gi* statistics, can automatically determine at what confidence level each spatial unit becomes a hotspot without human intervention. In addition, the proposed method is concise and can be easily implemented on different GIS platforms. Therefore, the method has great potential for the rapid detection of large-scale traffic accident blackspots. Based on the OHSA results, an RBT index is proposed to measure the road traffic safety level of each city. To verify its practicability, this study performed a binary correlation analysis. The results show that there is a strong correlation between the RBT index and the number of traffic accidents. Finally, several recommendations for improving the road safety level and reducing traffic accidents are provided to policy makers and traffic engineers:

- Give priority to measures of reducing motor vehicle traffic accidents. According to Table 1, the number of motor vehicle accidents (MMA) is the largest, with a total of 8012, accounting for 65.7% of the annual total. Motor vehicle accidents black spots can be easily found in most areas, as seen in Figure 4f. Enlarging resource inclination disposition in the black spot area to reduce motor vehicle accidents is the task of each city.
- Determine the appropriate analysis scale. The optimal scalar distance should be determined with full consideration of the travel distance of the target traffic mode. The results of incremental spatial autocorrelation imply that the optimal scalar distances are different for various types of accidents. Merely using the entire accident dataset as the model input without considering the accident type may produce unreasonable distance bands. In turn, this may lead to insignificant feature identification or loss of data continuity.
- Implement more comprehensive optimization measures in the central city and vulnerable communities. The central city experiences more severe challenges regarding traffic safety compared to satellite cities. Moreover, the OHSA method can identify vulnerable communities. Various types of accidents tend to be concentrated in these vulnerable communities. Policy makers and traffic engineers are expected to give higher attention to vulnerable communities or prioritize the implementation of relevant optimization measures from the aspects of policy, road design, road management and traffic planning.
- Implement tailored optimization measures in the cities that have fixed types of blackspots. For example, one of the important findings of this study is non-motor vehicles are more likely to be responsible for traffic accidents in Xide, as seen in Figure 4a,c. If the optimization measures related to motor vehicles are blindly implemented without considering the negative impact of non-motor vehicles, the number of traffic accidents in Xide will not be significantly reduced even if the local financial and human resources are exhausted. Therefore, for such cities, it is necessary to apply the OHSA method to identify blackspots for each accident type and then enforce tailored traffic safety management measures.

One limitation of this study is that the geographic boundary areas (e.g., traffic cells, fishnet grids and hexagonal grids) are used as the basic mapping spatial unit. The shape of the blackspot could be confined to the shape of the pre-defined spatial unit. In an extension of this study, more comprehensive data can be collected to analyze the relationship between the number of traffic accidents and built environment or land use within a blackspot area. The adaptability could be better verified if we obtain the data of other areas in the future.

## Figures and Tables

**Table 1:** A summary of the crash dataset.

Classification by Accident Participants (Count)								
Type	UA	NNA	NPA	MNA	MMA	MPA	Sum	%
**MA**	1283	313	161	1551	7863	285	11,456	94.0
**OA**	59	59	53	258	141	123	693	13.9
**SA**	8	0	1	9	8	12	38	0.3
**Sum**	1350	372	215	1818	8012	420	12,187	100
**%**	11.1	3.1	1.8	14.9	65.7	3.4	100	\

## Data Availability

Not applicable.

## Abbreviations

The following abbreviations are used in this manuscript:

Abbreviation	Description
OHSA	Optimized Hot Spot Analysis
KDE	Kernel Density Estimation
SDE	Standard Deviational Ellipses
UA	Unilateral accidents
MMA	Motor vehicle accidents
NNA	Non-motor vehicle accidents
NPA	Non-motor vehicle and pedestrian accidents
MNA	Motor vehicle and non-motor vehicle accidents
MA	Minor accidents
OA	Ordinary accidents
SA	Serious accidents
MPA	Motor vehicle and pedestrian accidents

## References

[1] National Bureau of Statistics (2020). Traffic Fatalities in China. https://app.mps.gov.cn.

[2] National Highway Traffic Safety Administration (2020). Fatality Analysis and Reporting System (FARS). https://www.nhtsa.gov/file-downloads?p=nhtsa/downloads/FARS/.

[3] Jiang C., Tay R., Lu L. (2021). A skewed logistic model of two-unit bicycle-vehicle hit-and-run crashes. Traffic Inj. Prev..

[4] Kuo P., Lord D. (2020). Applying the colocation quotient index to crash severity analyses. Accid. Anal. Prev..

[5] Zheng Z., Lu P., Lantz B. (2018). Commercial truck crash injury severity analysis using gradient boosting data mining model. J. Saf. Res..

[6] Jiang C., Lu L., Chen S., Lu J.J. (2016). Hit-and-run crashes in urban river-crossing road tunnels. Accid. Anal. Prev..

[7] Li G., Yang Z., Pan Y., Ma J. (2022). Analysing and modelling of discretionary lane change duration considering driver heterogeneity. Transp. B Transp. Dyn..

[8] Li G., Yang Z., Yu Q., Ma J., Fang S. (2021). Characterizing Heterogeneity among Merging Positions: Comparison Study between Random Parameter and Latent Class Accelerated Hazard Model. J. Transp. Eng. Part A Syst..

[9] Cheng W., Washington S.P. (2005). Experimental evaluation of hotspot identification methods. Accid. Anal. Prev..

[10] Environmental Systems Research Institute (2021). How Hot Spot Analysis (Getis-Ord Gi*) Works. https://pro.arcgis.com/en/pro-app/latest/tool-reference/spatial-statistics/h-how-hot-spot-analysis-getis-ord-gi-spatial-stati.htm.

[11] Kwon O.H., Park M.J., Yeo H., Chung K. (2013). Evaluating the performance of network screening methods for detecting high collision concentration locations on highways. Accid. Anal. Prev..

[12] Benedek J., Ciobanu S.M., Man T.C. (2016). Hotspots and social background of urban traffic crashes: A case study in Cluj-Napoca (Romania). Accid. Anal. Prev..

[13] Chen Y., Wang K., Zhang Y., Shi Q. (2021). Identification of black spots on highways using fault tree analysis and vehicle safety boundaries. J. Transp. Saf. Secur..

[14] Cui H., Dong J., Zhu M., Li X., Wang Q. (2022). Identifying accident black spots based on the accident spacing distribution. J. Traffic Transp. Eng. (Engl. Ed.).

[15] Yuan T., Zeng X., Shi T. (2020). Identifying Urban Road Black Spots with a Novel Method Based on the Firefly Clustering Algorithm and a Geographic Information System. Sustainability.

[16] Vandenbulcke G., Thomas I., Panis L.I. (2014). Predicting cycling accident risk in Brussels: A spatial case–control approach. Accid. Anal. Prev..

[17] Hu Y., Zhang Y., Shelton K.S. (2018). Where are the dangerous intersections for pedestrians and cyclists: A colocation-based approach. Transp. Res. Part C Emerg. Technol..

[18] Kockelman K.M., Kweon Y. (2002). Driver injury severity: An application of ordered probit models. Accid. Anal. Prev..

[19] Ye F., Lord D. (2014). Comparing three commonly used crash severity models on sample size requirements: Multinomial logit, ordered probit and mixed logit models. Anal. Methods Accid. Res..

[20] Li L., Zhu L., Sui D.Z. (2007). A GIS-based Bayesian approach for analyzing spatial–temporal patterns of intra-city motor vehicle crashes. J. Transp. Geogr..

[21] Wen X., Xie Y., Wu L., Jiang L. (2021). Quantifying and comparing the effects of key risk factors on various types of roadway segment crashes with LightGBM and SHAP. Accid. Anal. Prev..

[22] Debrabant B., Halekoh U., Bonat W.H., Hansen D.L., Hjelmborg J., Lauritsen J. (2018). Identifying traffic accident black spots with Poisson-Tweedie models. Accid. Anal. Prev..

[23] Wang D., Ding W., Lo H., Stepinski T., Salazar J., Morabito M. (2013). Crime hotspot mapping using the crime related factors—A spatial data mining approach. Appl. Intell..

[24] Yuill R.S. (1971). The Standard Deviational Ellipse; An Updated Tool for Spatial Description. Geogr. Ann. Ser. B Hum. Geogr..

[25] Anderson T.K. (2009). Kernel density estimation and K-means clustering to profile road accident hotspots. Accid. Anal. Prev..

[26] Ryder B., Gahr B., Egolf P., Dahlinger A., Wortmann F. (2017). Preventing traffic accidents with in-vehicle decision support systems—The impact of accident hotspot warnings on driver behaviour. Decis. Support Syst..

[27] Shi Z., Pun-Cheng L.S.C. (2019). Spatiotemporal Data Clustering: A Survey of Methods. ISPRS Int. J. Geo-Inf..

[28] Li Y., Abdel-Aty M., Yuan J., Cheng Z., Lu J. (2020). Analyzing traffic violation behavior at urban intersections: A spatio-temporal kernel density estimation approach using automated enforcement system data. Accid. Anal. Prev..

[29] Xie Z., Yan J. (2008). Kernel Density Estimation of traffic accidents in a network space. Comput. Environ. Urban Syst..

[30] Brunsdon C., Corcoran J., Higgs G. (2007). Visualising space and time in crime patterns: A comparison of methods. Comput. Environ. Urban Syst..

[31] Zhang K., Wang Z. (2022). LTPP data-based investigation on asphalt pavement performance using geospatial hot spot analysis and decision tree models. Int. J. Transp. Sci. Technol..

[32] Ord J.K., Getis A. (1995). Local Spatial Autocorrelation Statistics: Distributional Issues and an Application. Geogr. Anal..

[33] Environmental Systems Research Institute (2021). Optimized Hot Spot Analysis (Spatial Statistics). https://pro.arcgis.com/en/pro-app/latest/tool-reference/spatial-statistics/optimized-hot-spot-analysis.htm.

[34] Lu P., Bai S., Tofani V., Casagli N. (2019). Landslides detection through optimized hot spot analysis on persistent scatterers and distributed scatterers. ISPRS J. Photogramm. Remote Sens..

[35] Ma C., Pu R., Downs J., Jin H. (2022). Characterizing Spatial Patterns of Amazon Rainforest Wildfires and Driving Factors by Using Remote Sensing and GIS Geospatial Technologies. Geosciences.

[36] Bai P., Schipperijn J., Rosenberg M., Christian H. (2022). Where are preschoolers active in childcare centers? A hot-spot analysis using GIS, GPS and accelerometry data. Child. Geogr..

[37] Purwanto P., Utaya S., Handoyo B., Bachri S., Astuti I.S., Utomo KS B., Aldianto Y.E. (2021). Spatiotemporal Analysis of COVID-19 Spread with Emerging Hotspot Analysis and Space–Time Cube Models in East Java, Indonesia. ISPRS Int. J. Geo-Inf..

[38] Xiao Y., Ouyang Z., Xu W., Xiao Y., Zheng H., Xian C. (2016). Optimizing hotspot areas for ecological planning and management based on biodiversity and ecosystem services. Chin. Geogr. Sci..

[39] Environmental Systems Research Institute (2021). Incremental Spatial Autocorrelation (Spatial Statistics). https://pro.arcgis.com/zh-cn/pro-app/latest/tool-reference/spatial-statistics/incremental-spatial-autocorrelation.htm.

[40] Liangshan Big Data Center (2022). Liang Shan Zhou Public Data Open Website. https://data.lsz.gov.cn/oportal/index.

[41] Zaït M., Messatfa H. (1997). A comparative study of clustering methods. Future Gener. Comput. Syst..

[42] Songchitruksa P., Zeng X. (2010). Getis–Ord Spatial Statistics to Identify Hot Spots by Using Incident Management Data. Transp. Res. Rec..

[43] Yang C., Chen M., Yuan Q. (2021). The application of XGBoost and SHAP to examining the factors in freight truck-related crashes: An exploratory analysis. Accid. Anal. Prev..

[44] Mane A.S., Pulugurtha S.S. (2018). Influence of on-network, traffic, signal, demographic, and land use characteristics by area type on red light violation crashes. Accid. Anal. Prev..

[45] Parsa A.B., Movahedi A., Taghipour H., Derrible S., Mohammadian A.K. (2020). Toward safer highways, application of XGBoost and SHAP for real-time accident detection and feature analysis. Accid. Anal. Prev..

